# Risk factors for necrotizing enterocolitis in neonates: a systematic review of prognostic studies

**DOI:** 10.1186/s12887-017-0847-3

**Published:** 2017-04-14

**Authors:** Noor Samuels, Rob A. van de Graaf, Rogier C. J. de Jonge, Irwin K. M. Reiss, Marijn J. Vermeulen

**Affiliations:** Department of Pediatrics, Division of Neonatology, Erasmus MC, Room SH-3005, P.O. Box 2060, 3015 CN Rotterdam, The Netherlands

**Keywords:** necrotizing enterocolitis, intestinal perforation, preterm, neonatal intensive care unit, risk factors, prognosis, epidemiology

## Abstract

**Background:**

Necrotizing enterocolitis (NEC) is a severe multifactorial disease in preterm neonates associated with high morbidity and mortality. Better insight into prognostic values of the many reported factors associated with NEC is needed to enable identification of neonates at risk for NEC. The aim was to systematically review the literature to identify independent risk factors for NEC from the literature.

**Methods:**

Medline, Cochrane, Embase, Pubmed and Google Scholar were searched systematically for cohort studies reporting prognostic factors for NEC in neonates using multivariable analysis. Studies were scored with the Quality In Prognosis Studies tool (QUIPS).

**Results:**

From 5154 initial hits, 14 prognostic studies were included, with various designs. Study quality was rated high in three studies, moderate or low in the 11 others. Significant prognostic factors for NEC reported in at least two studies were: low birth weight, small for gestational age, low gestational age, assisted ventilation, premature rupture of membranes, black ethnicity, sepsis, outborn, hypotension (all increased risk), surfactant therapy (conflicting results) and cesarean section (lower risk). Meta-analysis was considered not feasible.

**Conclusion:**

High quality studies on prognostic factors for NEC are rare. Several prognostic factors, that are not necessarily causal, are associated with NEC. High quality prognostic research is necessary to establish the predictive values of these factors.

**Electronic supplementary material:**

The online version of this article (doi:10.1186/s12887-017-0847-3) contains supplementary material, which is available to authorized users.

## Background

Necrotizing enterocolitis (NEC) is one of the most severe complications of preterm birth occurring in 5–10% of very low birth weight infants [1, 2]. Although more and more (extremely) preterm infants survive, the number of deaths attributed to NEC has been increasing [3]. Mortality rates ranging from 15% to 30% have been reported [4]. Surgical treatment is often needed, and survivors are at increased risk for poor long-term growth and neurodevelopmental impairment [5]. Despite preventive strategies such as prenatal glucocorticoid administration, breast feeding, use of donor milk and probiotic supplementation, NEC is still relatively common in most neonatal intensive care units (NICUs) [6–10].

NEC is difficult to predict in individual cases. The etiology is complex and multifactorial, including genetic predisposition, intestinal immaturity, imbalance in microvascular tone, abnormal microbial colonization and highly immune-reactive intestinal mucosa [1]. A common inflammatory pathway leads to intestinal ischemia, pneumatosis, necrosis and eventually perforation [11].

Many observational studies have reported clinical and non-clinical risk factors associated with NEC, but the prognostic value usually is unclear. Most of these studies were not designed to answer prognostic questions properly [12]. To identify independent risk factors for a complex disease as NEC, a (preferably prospective) prognostic cohort design with multivariable analysis including multiple co-variates is considered most appropriate [12–14]. The aim of this study was to provide a systematic review of the literature on prognostic studies reporting on independent risk factors for NEC in neonates.

## Methods

### Study selection

This systematic review was guided by the PRISMA Statement, a 27 item checklist to improve the reporting of systematic reviews [15]. A search strategy was developed in collaboration with a clinical librarian to search *PubMed*, *Embase*, *Medline*, *Web-of-science*, *Cochrane* and *Google Scholar*. An initial search was conducted in January 2014 and updated in August 2016, using terms related to *necrotizing enterocolitis*, *intestinal perforation*, *neonates*, *birth weight*, *gestation*, *prediction, prognosis*, *epidemiology* and *risk factors*. The complete search strategy is reported as supplemental material (Additional file 1). References of included studies were checked for additional eligible studies.

- Studies were included for analysis if satisfying all following criteria: (1) full English written publications, (2) with a prospective or retrospective cohort study or nested case-control design (3) identifying (neonatal or non-neonatal) prognostic factors for NEC (primary or secondary outcome), (4) using multivariable data analysis including more than 2 co-variates, (5) in a study population of neonates/newborns, preterm infants, very low birth weight (VLBW) or extremely low birth weight (ELBW) infants. No explicit use of the term *prognostic* was required for inclusion.
- Excluded were studies (1) only reporting on associative models of one or two variables with NEC (also if adjustment for potential confounders was performed), or (2) focusing only on risk factors for other abdominal problems than NEC such as spontaneous focal intestinal perforation, viral enteritis and allergic colitis.

After duplicates had been removed, two independent reviewers (NS, RG) screened titles and abstracts on both inclusion and exclusion criteria. Articles identified as potentially eligible underwent a full text review. Any disagreements between the two reviewers concerning study selection, quality assessment and interpretation of results were discussed and resolved in consensus meetings with all authors.

### Quality assessment

The methodological quality of full text reports was independently assessed by the same researchers using the Quality In Prognosis Studies (QUIPS) tool [16]. The QUIPS tool assesses risk of bias in prognostic studies by rating each individual article in six domains: study participation, study attrition, measurement of prognostic factors, measurement of outcomes, measurement of confounding, and statistical analysis and reporting. As prognostic studies are designed to predict a specific outcome based on a combination of possible prognostic factors of equal interest, the domain of confounding was considered irrelevant. Therefore, an adapted QUIPS without items addressing confounding was used [17]. As NEC was assumed to be a short-term outcome in the included studies, items on long-term follow-up in the quality assessment were not included.

Quality points for a total of 17 items in five domains were assigned to each study, adding up to a total score of 75 points maximum. Domain items were scored as high when sufficient information concerning the risk of bias was present and the estimated risk of bias was considered low. Items were scored as moderate when the article provided insufficient information about this domain and low quality when an item was not reported or was not reported clearly or the estimated risk of bias was considered high. The quality of the studies was ranked high if ≥60 points (≥80% of the maximum score); moderate if 45–59 points (≥60% and <80% of the maximum score); and low if <45 points (<60% of maximum score) were given as described previously [17].

### Data extraction and reporting

A standardized form was used to guide and document data extraction systematically. The following data were extracted: study characteristics (publication characteristics, study design, method of analysis, number of subjects, type and number of prognostic factors, outcomes of interest); patient characteristics (gestational age, birth weight, gender); and strength of association (relative risks (RR) and odds ratios (OR)). To restrict the data to those of most clinical interest, we only present details on statistically significant prognostic factors (*p* < 0.05) that were reported in at least two studies.

## Results

### Search and inclusion

**Fig. 1:**
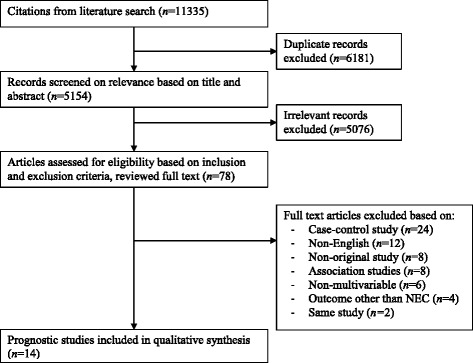
Flow chart of the systematic literature search

The literature search yielded a total of 11,335 studies (Fig. 1). After removing duplicates 5154 articles remained of which titles and abstracts were screened. Full texts of 78 articles were retrieved after assessment for eligibility. A total of 14 articles met the selection criteria and were included in the study. No additional eligible studies were identified through bibliographic review of the included studies.

### Study characteristics

The quality assessment results are listed in Table 1. Three of the included studies were of high quality, 11 were of moderate quality and none of low quality. Details of the included studies are presented in Table 2. Definitions of study population and outcome varied. Ten studies had a retrospective study design and four studies a prospective study design. In seven studies the study population consisted of VLBW infants, defined by birth weight below 1500 g. One study included neonates with intrauterine growth retardation (IUGR) and two studies neonates admitted to the NICU. In four studies the population was defined by gestational age: below 33 weeks, 23–34, 23–32 and 23–36 weeks. The outcome measure was often not clearly described and included NEC Bell stages I and II in one study, and stages II and III in all others.

**Table 1:** Results of quality assessment of studies on NEC related prognostic factors according to the QUIPS tool [17]

Study	Study participation (max. 15)	Study attrition (max. 15)	Prognostic factor measurement (max. 15)	Outcome measurement (max. 15)	Statistical analysis and reporting (max. 15)	Quality score (max. 75)
Gephart et al. (2014) [18]	15	5	12.5	15	15	62.5
Lee et al. (2016) [38]	15	5	12.5	12.5	15	60
Youn et al. (2015) [30]	15	5	12.5	12.5	15	60
Boo et al. (2012) [21]	15	5	12.5	10	15	57.5
Drenckpohl et al. (2010) [36]	15	5	10	12.5	15	57.5
Guthrie et al. (2003) [23]	15	5	10	12.5	15	57.5
Yee et al. (2012) [27]	15	5	12.5	10	15	57.5
Gagliardi et al. (2008) [28]	15	5	12.5	7.5	15	55
Manogura et al. (2008) [39]	15	5	10	10	15	55
Yamoto et al. (2016) [26]	15	5	10	10	15	55
Faustini et al. (2003) [37]	13.5	5	10	12.5	12.5	53.5
Carter et al. (2008) [22]	15	5	10	10	12.5	52.5
Luig et al. (2004) [25]	15	5	10	7.5	15	52.5
Uauy et al. (1991) [24]	12	5	10	10	12.5	49.5

*NEC* necrotizing enterocolitis, *QUIPS* quality in prognosis studies

**Table 2:** Methodologic characteristics of the included prognostic studies on risk factors for NEC

Author	Country (year)	Design	Type of analysis	*N* included	Male (%)	Study population	Outcome	NEC type	Significant prognostic factors from multivariable analysis with *p* < 0.05
Boo et al. (2012) [21]	Malaysia (2007)	Retrospective	Multivariable	3601	52%	VLBW (≤1500 g)	NEC	II, III	Maternal age, BW, surfactant, intrapartum antibiotics, indomethacin, surfactant, congenital pneumonia
Carter et al. (2008) [22]	United States (unknown)	Retrospective	Multivariable	134	54%	GA <35 weeks, BW <1500 g or requiring mechanical ventilation at birth	NEC	II, III	Number of infections, ventilation
Drenckpohl et al. (2010) [36]	United States (2002–2008)	Retrospective	Multivariable	324	54%	GA 23–36 weeks	NEC	II, III	Ethnicity, PROM, sepsis, H2 blockers in TPN
Faustini et al. (2003) [37]	Italy (1999)	Retrospective	Multivariable	221	49%	Live births of the university hospital in Rome	NEC	I, II	Any neonatal pathological condition, first time feeding with formula, any invasive procedure
Gagliardi et al. (2008) [28]	Italy (1999–2002)	Prospective	Multivariable	2035	51%	VLBW (<1500 g), GA 23–37 weeks	NEC-medical, NEC-surgical	II, III	Assisted ventilation, PDA, surfactant, Late-onset sepsis
Gephart et al. (2014) [18]	United States (2007–2011)	Retrospective	Multivariable (Prediction model)	35,013	50%	BW <1500 g and GA < 36 weeks	NEC-surgical, NEC-medical	II, III	GA, outborn, ethnicity, dopamine, dobutamine or milrinone combined with hypotension, metabolic acidosis, probiotics, late sepsis, PRBC transfusion, 2 or more positive cultures (blood, urine, other), human milk at day 7 and 14 of life, unit NEC rate
Guthrie et al. (2003) [23]	United States (1998–2000)	Retrospective	Multivariable	15,072	53%	GA 23–34 weeks	NEC-surgical, NEC-medical	II, III	BW, antenatal glucocorticoids, umbilical vessel catheterization, assisted ventilation, type of delivery, exposed both glucocorticoids and indomethacin during first week of life, Apgar score 5 min.
Lee et al. (2016) [38]	South Korea (2003–2014)	Retrospective	Multivariable	354	53%	GA 23–31(+6) weeks	NEC	II, III	Maternal blood NLR, multiparity, BW, GA
Luig et al. (2004) [25]	Australia (1994–1999)	Retrospective	Multivariable	4649	55%	All NICU admissions	NEC	II, III	Placental abruption, GA (weeks), SGA, year of birth, hyaline membrane disease, hypertensive disease of pregnancy
Manogura et al. (2008) [39]	United States (1997–2006)	Prospective	Multivariable	404	^a^	Neonates with suspected IUGR	NEC	II, III	BW, base deficit
Uauy et al. (1991) [24]	United States (1988–1989)	Prospective	Multivariable	2681	^a^	Infants admitted to one of the network centers <1500 g	NEC	II, III	Center of birth, ethnicity/gender, BW, maternal haemorrhage, duration of ROM, cesarean section
Yamoto et al. (2016) [26]	Japan (2006–2015)	Retrospective	Multivariable	323	53%	BW <1000 g	NEC, FIP, MRI	II, III	Gestational age of <26 weeks, severe cardiac malformations, not received EAP
Yee et al. (2012) [27]	Canada (2003–2008)	Retrospective	Multivariable	16,669	54%	GA <33 weeks	Early-Onset NEC, Late-Onset NEC	II, III	GA, SGA, outborn/inborn, congenital anomalies, narcotic use in <3 days, postnatal glucocorticoids
Youn et al. (2015) [30]	Korea (2013–2014)	Prospective	Multivariable	2326	50%	VLBW (<1500 g)	NEC	II, III	hypotension ≤1 week

*BW* birth weight, *EPO* erythropoietin, *FIP* focal intestinal perforation, *GA* gestational age, *IVH* intraventricular hemorrhage, *MRI* meconium-related ileus, *NCPAP* nasal continuous positive airway pressure, *NEC* necrotizing enterocolitis, *NICU* neonatal intensive care unit, *NLR* neutrophil-lymphocyte ratio, *PDA* patent ductus arteriosus, *PRBC* packed red blood cell, *(P)ROM* (premature) rupture of membranes, *RDS* respiratory distress syndrome, *SGA* small for gestational age, *SNAP* score for neonatal acute physiology, *TPN* total parental nutrition, *VLBW* very low birth weight

Footnotes: ^a^ not available

### Prognostic factors

The 14 included studies described 43 statistically significant risk factors for NEC identified by multivariable analysis. Eleven of these factors were significantly associated with NEC in at least two studies (Table 3). None of the prognostic factors were assessed in all studies. Of the 11 reproducible factors, the following were associated with an increased risk of NEC: small for gestational age, low gestational age, assisted ventilation, sepsis, hypotension, PROM, black ethnicity and outborn status (Table 4). The factor low birth weight was associated differently with NEC in 5 studies. The association with surfactant therapy also showed contrasting directions. Birth by cesarean section was associated with a decreased risk of NEC.

**Table 3:** Summary of significant prognostic factors for NEC by high, moderate and low quality studies

Prognostic factor	High quality	Moderate quality	Low quality
Birth weight	1×	4×	-
Gestational age (weeks)	1×	3×	-
Sepsis	1×	2×	-
Ethnicity	1×	2×	-
Hypotension	1×	1×	-
Outborn	1×	1×	-
Assisted ventilation	-	3×	-
Cesarean section	-	2×	-
PROM	-	2×	-
Small for gestational age	-	2×	-
Surfactant	-	2×	-

The table shows the statistically significant prognostic factors reported in at least two studies. The quality of the studies was ranked high if ≥60 points (≥80% of the maximum score), moderate if 45–59 points (≥60% and <80% of the maximum score) and low if <45 points (<60% of maximum score) were given. Using the QUIPS tool

*NEC* necrotizing enterocolitis, *PDA* patent ductus arteriosus, *PROM* premature rupture of membranes, *RDS* respiratory distress syndrome, *QUIPS* quality in prognosis studies

**Table 4:** Prognostic factors associated with NEC reported in at least two studies

First author:	Boo	Carter	Drenckpohl	Faustini	Gagliardi	Gephart	Guthrie	Lee	Luig	Manogura	Uauy	Yamoto	Yee	Youn
(Low) birth weight^b^	OR 0.999 [0.998,0.999]^c^	OR 1.001 [*p* = 0.164]^d,e^	OR 1 [1.000,1.001]	.	.	.	*p* < 0.001^f^	OR 0.07 [0.01,0.53]^g^	.	*p* < 0.001^f^	OR 0.999 [*p* < 0.001]^c,d,h^	.	.	.
Gestational age (weeks)^b^	.	.	.	.	.	OR 2.37 [1.78,3.16]^i^	.	OR 1.14 [0.85,1.54]^g^	OR 0.82 [0.77,0.89]^g^	.	.	OR 19.32 [3.27,370.43]^j^	OR 0.84 [0.81,0.87]^g^	.
Sepsis	.	.	OR 4.98 [2.2,11.27]^a^	.	OR 5.38 [2.86,10.14]^k^	OR 1.49 [1.30,1.72]^k^	.	OR 1.41 [0.42,4.67]^a^	.	.	.	.	.	OR 1.75 [0.71,2.86]^l^
Ethnicity	.	OR 0.571 [*p* = 0.317]^d,m^	OR 0.36 [0.17,0.78]^n^	.	.	OR 1.22 [1.09,1.35]^m^	.	.	.	.	OR 1.68 [*p* < 0.001]^d,h,m^	.	.	.
Hypotension	.	.	.	.	.	OR 1.51 [1.36,1.69]^o^	.	.	.	.	.	.	.	OR 2.00 [1.001,3.999]^o^
Outborn	.	.	.	.	.	OR 1.31 [1.17,1.46]	.	.	.	.	.	.	OR 1.55 [1.31,1.83]	.
Assisted ventilation	.	OR 1.053 [*p* = 0.047]^d,a^	.	.	OR 2.71 [1.03,7.15]^a^	.	OR 3.5 [2.5,4.7]^p^	.	.	.	.	.	.	.
Cesarean section	.	.	.	.	.	.	OR 0.60 [0.50,0.80]	.	.	.	OR 0.60 [*p* < 0.001]^d,h^	.	.	.
PROM	.	.	OR 2.06 [1.02,4.16]	.	.	.	.	.	.	.	OR 1.10 [*p* < 0.009]^d,h^	.	.	.
Small for gestational age^b^	.	.	.	.	.	.	.	.	OR 1.97 [1.19,3.26]^a^	.	.	.	OR 1.35 [1.08,1.69]^q^	.
Surfactant	OR 1.59 [1.17,2.16]	.	.	.	OR 0.41 [0.19,0.90]	.	.	.	.	.	.	.	.	.

This table shows the strength and the direction of association of prognostic factors, with concomitant confidence intervals if available

*NEC* necrotizing enterocolitis, *OR* odds ratio, *PROM* premature rupture of membranes

Footnotes: ^a^ exact definition was lacking; ^b^ cutt-off values differs between studies; ^c^ < 1500 g; ^d^ no CI was given or could be calculated; ^e^ < 1500 g or requiring mechanical ventilation at birth; ^f^ OR increased with decreasing weight, no exact ratio was given; ^g^ calculated for increase of one unit of the continuous variable; ^h^ calculated by results presented in article; ^*i*^ < 28 weeks; ^j^ < 26 weeks; ^k^ late-onset sepsis; ^l^ combination of early- and late-onset sepsis; ^m^ black ethnicity; ^n^ white ethnicity; ^o^ inotropics requirement; ^p^ mechanical ventilation first day of life; ^q^ < 10th percentile for the given GA

Because of the diversity in study population, definition and incidence of outcome and type of analysis, the measures of associations need to be interpreted in the context of the study characteristics as reported in Table 2. Because of substantial heterogeneity in design, population, prognostic factors and outcomes of the included prognostic studies, it was not feasible to perform a meta-analysis.

## Discussion

This is the first systematic review of prognostic studies on risk factors for NEC in neonates. Only three of the 14 included studies scored high for methodological quality; all others scored moderate. This was mostly because of limited information on definition and measurement of the prognostic factor and on the outcome of NEC. Only one report was found on the development of a prediction model for the outcome of NEC [18].

We defined a *prognostic study design* based on several specific criteria suggested in the literature. Aiming to estimate the risk of developing a future clinical outcome (NEC) based on more than one (independent) characteristic, is considered the key feature of a prognostic study design [12–14]. Therefore we only included studies with a defined study population and multivariable analysis of variables of potential equal importance. We did not include trials or studies that focused on the association between NEC and a single risk factor. Although these studies may generate relevant hypotheses, these do not address prognostic questions from a clear prognostic research perspective [12].

### Risk factors for NEC

Low birth weight is the most commonly reported significant prognostic factor for NEC among neonates in the current literature, which is in line with large cohort studies describing the highest incidence of NEC among the infants with the lowest birth weights [19, 20]. Interestingly, the clinical relevance of birth weight as an independent prognostic factor for NEC is questionable with odds ratios (ORs) ranging between 0.999–1.001 [21–24]. Presumably, low gestational age or being small for gestational age are clinically more important. However, associations between NEC and these factors were only confirmed by multivariable analysis in four and two of the studies respectively [18, 25–27].

Also of interest, two studies showed a protective effect of cesarean section for developing NEC (OR both 0.60) [23, 24]. The authors suggest that this is due to less stress during delivery, although they point out that selection bias may have occurred. Surfactant therapy proved a positive predictor for NEC in one study but a negative predictor in another [21, 28]. The authors of the latter study explained this by improvement of pulmonary function leading to less gut ischemia. Boo et al. reported that surfactant was administered only to infants with severe respiratory distress syndrome (RDS), suggesting that not surfactant but severe RDS was a significant risk factor for NEC [21]. Kliegman et al. studied RDS and NEC and concluded that neonatal hypoxia is not etiologically related to NEC [29]. They found that mild or no RDS was associated with an increased risk of NEC in contrast to severe RDS, however by performing only univariable analysis. It is unclear whether this can be explained by a protective effect of surfactant in the severe cases or by other factors such as different nutritional or antibiotic treatment. Assisted ventilation was also associated with an increased risk of NEC [22, 23, 28]. The question arises whether this reflects disease severity - as the sickest patients (with the highest risk for NEC) will need ventilation - or the mechanical ventilation itself, as disease severity (other than by birth weight and gestation age) was not adjusted for in all of these studies.

Gephart et al. were the only authors who described the development of a prediction model. Their model (GutCheck^NEC^) included 10 clinical risk factors based on a large neonatal dataset [18]. They found that hypotension requiring inotropic treatment was associated with an increased risk of NEC. Also, Youn et al. considered hypotension within a week of life, as an independent risk factor for NEC [30]. They suggest that the circulatory collapse in the first week of life may assault the gastrointestinal blood flow resulting in higher NEC incidence. Two studies showed neonates who were born outside of the hospital were at greater risk for developing NEC compared to inborn neonates, which was also included in the model of Gephart et al. [18, 27].

The only maternal or perinatal prognostic factors for which evidence in the prognostic literature was found were PROM, cesarean section and being inborn. Interestingly, for commonly assumed clinical neonatal risk factors for NEC such as umbilical lines, red cell transfusions, H2 blockers, and (high osmolar) formula feeding no prognostic evidence was found [20, 31–35]. These factors showed no significant associations in prognostic studies, or have only been reported in studies without a prognostic design.

White ethnicity was associated with a lower risk for NEC compared to black ethnicity in multivariable analyses [18, 24, 36]. However, one study could not confirm these findings, probably due to the overwhelming effects of other factors in the multivariable model [22]. Another finding from two multivariable analyses is an association between diagnosis and treatment of sepsis and NEC, which was defined as blood culture proven late onset sepsis in one study and undefined sepsis prior to NEC in the other [28, 36].

### Interpretation of results

When interpreting the results, the following considerations should be taken into account. Firstly, most included studies were of limited quality and heterogeneous. The incidence of NEC stages II-III varied widely, probably due to differences in the selection of the study population and in NEC classification [18, 21–28, 30, 36–39].

Secondly, predictability is not synonymous to causality, although this is often inferred. This is most striking in studies that are unclear on or even ignore the temporality between exposure and onset of outcome. This was nicely pointed out by Patel et al., who studied the association between red blood cell (RBC) transfusions and NEC. They showed that severe anemia but not red blood cell (RBC) transfusion was associated with an increased risk of NEC and suggested that prevention of anemia may be more beneficial than minimizing RBC transfusions [40].

Discriminating etiologic and prognostic study designs is complex, especially because the methodological approaches overlap to some extent. Prognostic research focuses on the probability of a particular state of health whereas etiological research aims to assess the causal relationship between risk factors and outcome. Therefore every causal factor is a predictor but not every predictor is causally related to the outcome [12, 41].

Lastly, the problem of unreported negative findings even within a published report is worth mentioning. Not all studies described the total set of baseline variables included in the multivariable model. Univariable significant factors may have tested non-significant in multivariable models and left out of the final model. By not reporting non-significant factors, it remains impossible to rule out factors that are often assumed to be predictors for NEC. Also, the prognostic factors reported in this review, may have been non-significant in other reports.

### Strengths and limitations

Strengths of this review are the broad search strategy, the systematic rating of risk of bias using QUIPS and reporting of data according to PRISMA. Dretzke’s key points for the methodological approach for systematic review of prognostic factors were followed [42].

Nevertheless, several limitations may have influenced our study selection and results. Reliable identification of prognostic studies can be difficult especially in case of incomplete reporting and diversity of terminology [42]. Language bias may have occurred by excluding non-English articles. Earlier reported but arbitrarily set cut-off points for low, moderate and high quality in the QUIPS rating were used. Prognostic factors studied once were not summarized in this review but can also be relevant predictors of NEC. This concerned, for example, the only report on multivessel fetal Doppler imaging, by Manogura et al., which still may be relevant [39].

### Future perspectives

Well-designed prospective prognostic studies are needed with detailed reporting on definitions, methods and measurement of the prognostic factor and outcome. Special attention should be given to timing of the exposures in relation to the diagnosis of NEC. It would be interesting not only to focus on patient factors but also on maternal factors. Maternal lifestyle factors (such as smoking and obesity), morbidity (such as diabetes, preeclampsia, and chorioamnionitis) and prenatal medication (such as antibiotics and corticosteroids) may also be relevant risk factors for NEC [43–48]. A second step is the development and validation of a prediction model to quantify individual risk profiles and identify patients at risk. Until now this has only been performed by Gephart et al. who developed a model based on a large set of retrospective data. Their model still needs external validation in other neonatal populations to evaluate its general clinical usefulness. In our opinion development and validation of prospective prediction models are still necessary for preventive strategies and future reduction of the incidence of NEC.

## Conclusion

It is concluded that high quality studies on prognostic factors for NEC are rare. Several prognostic factors are associated with NEC, of which not all are necessarily causal. Ruling out factors is hampered by incomplete reporting. Future high quality prognostic (and predictive) research is necessary to enable clinicians to identify patients at high risk for NEC.

## Additional file

Additional file 1: Literature search strategy. (DOCX 118 kb)

## Abbreviations

ELBW: Extremely low birth weight
EPO: Erythropoietin
IVH: Intraventricular hemorrhage
NCPAP: Nasal continuous positive airway pressure
NEC: Necrotizing enterocolitis
NICU: Neonatal intensive care unit
OR: Odds ratio
PDA: Patent ductus arteriosus
PRISMA: Preferred reporting items for systematic reviews and meta-analyses
PROM: Premature rupture of membranes
QUIPS: Quality in prognosis studies
RDS: Respiratory distress syndrome
RR: Relative risk
SNAP: Score for neonatal acute physiology
TPN: Total parenteral nutrition
VLBW: Very low birth weight
