# Supplementary material for: Risk factors for necrotizing enterocolitis in neonates: a systematic review of prognostic studies
Source: BMC Pediatr. 2017 Apr 14;17:105. doi: 10.1186/s12887-017-0847-3 (PMC5391569; doi:10.1186/s12887-017-0847-3)
Supplement: Additional file 1: — Literature search strategy. (DOCX 118 kb) [file 12887_2017_847_MOESM1_ESM.docx]

# Additional file 1

# Risk factors for necrotizing enterocolitis in neonates: a systematic review of prognostic studies

# Noor Samuels^1^, Rob A. van de Graaf^1^, Rogier C.J. de Jonge^1^, Irwin K.M. Reiss^1^, Marijn J. Vermeulen^1^

**Short title:** Prognostic factors for necrotizing enterocolitis

**Affiliation:** ^1^Department of Pediatrics, division of Neonatology, Erasmus MC, Rotterdam, The Netherlands

# Literature search strategy

**Embase n=2800**

('necrotizing enterocolitis'/de OR 'necrotizing enteritis'/de OR 'intestine perforation'/de OR 'pseudomembranous colitis'/de OR ((necroti* NEAR/3 (enterocolitis OR enteritis)) OR (intestin* NEAR/3 perforat*) OR (pseudomembran* NEXT/1 colitis)):ab,ti) AND (newborn/de OR 'birth weight'/exp OR 'gestational age'/de OR prematurity/de OR 'newborn intensive care'/de OR (newborn* OR (new* NEXT/1 born*) OR neonat* OR preterm* OR gestation* OR 'birth weight' OR lbw OR vlbw OR elbw OR sga OR (small NEXT/2 date) OR prematur* OR baby OR babies OR nicu):ab,ti) AND (risk/exp OR etiology/exp OR epidemiology/exp OR 'epidemiological data'/exp OR 'necrotizing enterocolitis'/de/dm_et OR 'clinical feature'/de OR prediction/de OR (risk* OR pathogenes* OR etiolog* OR epidemiolog* OR incidence* OR associat* OR correlat* OR relation* OR feature* OR predict* OR (effect* NEXT/1 of)):ab,ti) NOT ([Review]/lim OR [Conference Abstract]/lim OR [Conference Paper]/lim OR [Letter]/lim OR [Note]/lim OR [Editorial]/lim OR [Short Survey]/lim OR [Erratum]/lim OR 'case report'/de) NOT ([animals]/lim NOT [humans]/lim)

**Medline (OvidSP) n=3047**

("Enterocolitis, Necrotizing"/ OR "Enterocolitis, Pseudomembranous"/ OR "Intestinal Perforation"/ OR ((necroti* ADJ3 (enterocolitis OR enteritis)) OR (intestin* ADJ3 perforat*) OR (pseudomembran* ADJ colitis)).ab,ti.) AND (exp "Infant, Newborn"/ OR "birth weight"/ OR "gestational age"/ OR "Intensive Care, Neonatal"/ OR "Intensive Care Units, Neonatal"/ OR (newborn* OR (new* ADJ born*) OR neonat* OR preterm* OR gestation* OR "birth weight" OR lbw OR vlbw OR elbw OR sga OR (small ADJ2 date) OR prematur* OR baby OR babies OR nicu).ab,ti.) AND (exp risk/ OR epidemiology/ OR "necrotizing enterocolitis"/et,ep OR (risk* OR pathogenes* OR etiolog* OR epidemiolog* OR incidence* OR associat* OR correlat* OR relation* OR feature* OR predict* OR (effect* ADJ 'of')).ab,ti.) NOT (Review OR congresses OR Letter OR Editorial OR Published Erratum).pt. NOT (case report*).ab,ti. NOT (exp animals/ NOT humans/)

**Cochrane n=231**

(((necroti* NEAR/3 (enterocolitis OR enteritis)) OR (intestin* NEAR/3 perforat*) OR (pseudomembran* NEXT/1 colitis)):ab,ti) AND ((newborn* OR (new* NEXT/1 born*) OR neonat* OR preterm* OR gestation* OR 'birth weight' OR lbw OR vlbw OR elbw OR sga OR (small NEXT/2 date) OR prematur* OR baby OR babies OR nicu):ab,ti) AND ((risk* OR pathogenes* OR etiolog* OR epidemiolog* OR incidence* OR associat* OR correlat* OR relation* OR feature* OR predict* OR (effect* NEXT/1 of)):ab,ti)

**Web-of-science n=2818**

TS=((((necroti* NEAR/3 (enterocolitis OR enteritis)) OR (intestin* NEAR/3 perforat*) OR (pseudomembran* NEAR/1 colitis))) AND ((newborn* OR (new* NEAR/1 born*) OR neonat* OR preterm* OR gestation* OR "birth weight" OR lbw OR vlbw OR elbw OR sga OR (small NEAR/2 date) OR prematur* OR baby OR babies OR nicu)) AND ((risk* OR pathogenes* OR etiolog* OR epidemiolog* OR incidence* OR associat* OR correlat* OR relation* OR feature* OR predict*)))

**PubMed publisher n=58**

((necrotising enter*[tiab] OR necrotizing enter*[tiab OR intestine perforat*[tiab] OR pseudomembranous colitis*[tiab])) AND ((newborn*[tiab] OR new born*[tiab] OR neonat*[tiab] OR preterm*[tiab] OR gestation*[tiab] OR birth weight*[tiab] OR lbw[tiab] OR vlbw[tiab] OR elbw[tiab] OR sga[tiab] OR prematur*[tiab] OR baby[tiab] OR babies[tiab] OR nicu[tiab])) AND ((risk*[tiab] OR pathogenes*[tiab] OR etiolog*[tiab] OR epidemiolog*[tiab] OR incidence*[tiab] OR associat*[tiab] OR correlat*[tiab] OR relation*[tiab] OR feature*[tiab] OR predict*[tiab])) AND publisher[sb]

**Google Scholar n=300**

("(necrotizing|necrotising) (enterocolitis|enteritis)"|"intestine perforation"|"pseudomembranous colitis") (newborn|newborns|premature|prematures|neonate|neonatal|nicu) (risk|etiology|epidemiology|prediction|incidence|association|associated|relation)
